# A combined computational-experimental approach to define the structural origin of antibody recognition of sialyl-Tn, a tumor-associated carbohydrate antigen

**DOI:** 10.1038/s41598-018-29209-9

**Published:** 2018-07-17

**Authors:** Ron Amon, Oliver C. Grant, Shani Leviatan Ben-Arye, Spandana Makeneni, Anita K. Nivedha, Tal Marshanski, Christoffer Norn, Hai Yu, John N. Glushka, Sarel J. Fleishman, Xi Chen, Robert J. Woods, Vered Padler-Karavani

**Affiliations:** 1 Department of Cell Research and Immunology, The George S. Wise Faculty of Life Sciences, Tel Aviv University, Tel Aviv, 69978 Israel; 2 Complex Carbohydrate Research Center, University of Georgia, Athens, 30606 GA USA; 3 Department of Biomolecular Sciences, Weizmann Institute of Science, Rehovot, 76100 Israel; 4 Department of Chemistry, University of California-Davis, Davis, CA USA

## Abstract

Anti-carbohydrate monoclonal antibodies (mAbs) hold great promise as cancer therapeutics and diagnostics. However, their specificity can be mixed, and detailed characterization is problematic, because antibody-glycan complexes are challenging to crystallize. Here, we developed a generalizable approach employing high-throughput techniques for characterizing the structure and specificity of such mAbs, and applied it to the mAb TKH2 developed against the tumor-associated carbohydrate antigen sialyl-Tn (STn). The mAb specificity was defined by apparent K_D_ values determined by quantitative glycan microarray screening. Key residues in the antibody combining site were identified by site-directed mutagenesis, and the glycan-antigen contact surface was defined using saturation transfer difference NMR (STD-NMR). These features were then employed as metrics for selecting the optimal 3D-model of the antibody-glycan complex, out of thousands plausible options generated by automated docking and molecular dynamics simulation. STn-specificity was further validated by computationally screening of the selected antibody 3D-model against the human sialyl-Tn-glycome. This computational-experimental approach would allow rational design of potent antibodies targeting carbohydrates.

## Introduction

Carbohydrates (glycans), as well as glycoproteins and glycolipids, are major cell surface components, and many monoclonal antibodies (mAbs) have been developed to target these molecules for various applications^1^. Changes in cellular glycosylation are common in cancer due to aberrant expression of glycosyltransferases, glycosidases, and transporters, as well as differences in the abundancy of carbohydrate building blocks^2–4^. Such alterations can result in unique antigenic glycans referred as Tumor-Associated Carbohydrate Antigens (TACAs). TACAs are used clinically as disease markers, for example, sialyl Lewis a (sLe^a^) is used for staging of pancreatic cancer^5^. Additionally, targeting TACA epitopes has become significant for managing various human cancers^6,7^. An antibody against the ganglioside GD2 has shown beneficial effects in neuroblastoma treatment^8^ and several other anti-carbohydrate antibodies are currently in clinical trials^1^. Although mAbs are clinically important tools, antibodies against carbohydrates tend to have low affinity^9^ and complex or mixed specificity^10^. In addition, the recognition of the glycan antigen can depend on glycan density, valency, presentation, and flexibility^11^. The paucity of high affinity and/or high specificity antibodies against carbohydrate targets is a crucial limitation in exploiting glycans as disease markers or therapeutic targets^12^.

The disaccharide Neu5Acα2–6GalNAcα1-*O*-Ser/Thr is a common short *O*-glycan TACA conjugated to proteins, also referred to as sialyl-Tn (STn; AcSTn). While this is a rare antigen or occluded in normal tissues due to further *O*-acetylation of the sialic acid (Sia)^3^, it is present in more than 80% of human carcinomas^13^, including those of the pancreas, breast, prostate, ovaries and colorectum^14,15^. Cells expressing the STn antigen have reduced adherence to extracellular matrix components, and show higher migration rates, thus promoting metastasis^16^. Moreover, the presence of STn is associated with decreased overall survival, and therefore has been the target for various immunotherapeutic research efforts^13^. There have been several unsuccessful attempts to develop a clinical viable vaccine against this antigen, including Theratope for human breast cancer patients that had induced STn-specific IgGs. Phase-III vaccine trial was carried out but largely failed because the participants had not been pre-evaluated for expression of STn^13^. In the Database of Anti-Glycan Reagents (DAGR; https://ccr2.cancer.gov/resources/Cbl/Tools/Antibody/), 14 different mAbs targeting STn are listed, albeit generally of low specificity. Some of these antibodies bind STn with either terminal *N*-acetylneuraminic acid (AcSTn; Neu5Acα2–6GalNAcαR) or *N*-glycolylneuraminic acid (GcSTn; Neu5Gcα2–6GalNAcαR), while another antibody did not bind any of these antigens on a glycan microarray^15^. Thus, improving the specificity of anti-STn antibodies is key for their potential use as tumor therapeutic and diagnostic (theranostics). Rational design can be used to improve an existing mAb, and for that purpose it is first imperative to define the structural features of the mAb binding region by co-crystallizing the antibody binding fragment (Fab) with the glycan antigen. Co-crystals of antibodies complexed with the corresponded TACA were generated for Le^x^, Le^y^ and Tn antigens^9^. Yet obtaining such Fab-antigen complex co-crystals is not always straightforward, and even more challenging in the case of anti-carbohydrate antibodies, due to their general low affinity^9^ and the plasticity of glycans. An alternative approach is to use computational modeling of the antibody, the glycan epitope, and their antibody-glycan binding interactions. However, computational approaches often lead to multiple plausible models, and orthogonal experimental data is essential for selecting the most likely model.

The relatively conserved structure of antibody domains, combined with the limited number of canonical 3D structures of the mAb hypervariable loops in the complementary determining regions (CDRs) makes it possible to predict the 3D structure of an antibody with reasonable accuracy^17–19^. Models of the antibody Fab can then be employed for automated ligand docking, using either experimental or theoretical structures of the glycans^20^. During docking, the small molecule ligand is generally permitted to be flexible while the protein receptor is often kept rigid, although a subset of protein side chains may be allowed flexibility. The accuracy of glycan docking can be enhanced if their unique conformational preferences are considered in the docking protocol^21^. Nevertheless, the accuracy (both in terms of the 3D pose of the ligand in the binding site, and its theoretical interaction energy) of computational docking alone is highly variable, and ideally the selection of the optimal theoretical antigen-antibody docking pose should be reinforced by experimental data^22^.

TKH2 is a unique anti-STn mAb, generated by immunization of mice with ovine submaxillary mucins^23^. This antibody binds various human cancer cells, such as lung, colon, breast, stomach and pancreas^23^. Unlike several other anti-STn antibodies^15^, the specificity of TKH2 antibody has been examined only against a limited set of glycans^23^ but not by more systematic glycan microarray assays. Here we developed an experimental-computational approach to investigate the specificity of anti-carbohydrate antibodies using the TKH2 as a model system. First, we obtained the antibody sequence by molecular cloning, and used it for antibody homology modelling and Molecular Dynamics (MD) simulations followed by computational docking of the glycan antigen. Subsequently, we investigated the antibody specificity by a sialoglycan microarray and alanine mutagenesis scanning, then together with STD-NMR defined the practical optimal antibody-antigen interactions. Computational grafting of 86 STn-related carbohydrate antigens of the human glycome database on the validated 3D antibody model further demonstrated the high specificity of the TKH2 antibody to the STn target. This approach can be used to explicitly elucidate antibody-antigen interactions that can be further used to improve the specificity of existing anti-carbohydrates antibodies to ultimately increase the use of such antibodies as theranostic tools.

## Results

### Obtaining VH and VL sequences of TKH2, an anti-STn monoclonal antibody

The anti-STn mAb TKH2^23^ holds great promise as a cancer theranostic tool (e.g. for therapy, diagnosis and monitoring of sTn-positive lesions) and was therefore selected for in-depth analysis to decipher its interaction with STn, and to determine its specificity using both various computational and experimental tools. To establish a computational model of the antibody, we determined the amino acid sequences of the mAb’s heavy and light chains variable domains (VH and VL; Supplementary Fig. 1a), which were then cloned and expressed with a human IgG Fc for further examination (Supplementary Fig. 1b).

For this purpose, we obtained the BM-4 hybridoma (ATCC), total RNA was extracted followed by cDNA synthesis and subsequently VH and VL fragments were amplified from the cDNA in a first-round PCR using appropriate primers (Supplementary Fig. 1b). For VH amplification, we used primers for mouse IgG1 since TKH2 was reported to be an IgG1^23^. For the kappa chain, the PCR amplification resulted in three similar-sized products and one larger product (data not shown), while no products were amplified in lambda VL PCR. Subsequently, the first-round PCR products were purified and sequenced (Supplementary Table 1a).

To confirm the identity of the obtained sequences, we used IgBLAST (http://www.ncbi.nlm.nih.gov/igblast/) against mouse Ig germline sequence database that also provides the translated sequence within the context of an antibody (Supplementary Table 1b,c), either according to the IMGT numbering system (http://www.imgt.org/) or KABAT numbering system (http://www.bioinf.org.uk/abs/). IgBLAST analysis of the heavy chain VH sequence aligned it to the IGHV2-3*01 mouse Ig germline gene sequence from the IMGT database. Thus, VH allele is closely related to family #2 and the germline gene to 3*01. The alignment also revealed several mutations in the query sequence compared to the germline sequence, including in the CDR regions, suggesting affinity maturation of the antibody. We then sequenced three of the PCR amplified light chain VL fragments and compared them by multiple sequence alignment (MSA) using ClustalW2 (http://www.ebi.ac.uk/Tools/msa/clustalw2) revealing two light chain variants. Since the hybridoma is in fact a fusion of two cells: the antibody secreting B cell and the immortalized myeloma, these two variants likely represented each one of them. In this specific hybridoma, the antibody-secreting B cell was fused to an SP2/0 myeloma cell^23^ that is known to express an aberrant non-functional kappa light chain^24,25^. Comparing the SP2/0 sequence (GenBank: M35669.1) with the obtained sequences by MSA revealed that two of them indeed represented the myeloma, while the third sequence likely represented the antibody VL (Supplementary Table 1a).

IgBLAST tool was used to align this sequence to mouse Ig germline, and the amino acid sequence of the VL (Supplementary Table 1b) was obtained. VL seemed to belong to the IGKV4-59*01 family and mutations were also observed compared to the germline sequence. The VH fragment was further amplified in a second-round nested PCR and these products were amplified again in a third nested PCR. Similarly, the VL fragment was amplified in second nested PCRs. Final products of VH and VL nested PCR rounds introduced restriction sites in both termini of the variable sequences, to facilitate cloning of the variable segments into expression vectors (Supplementary Fig. 1b). The PCR products were purified from gel, and sequence analysis validated that no mutation in the VH/ VL sequences were caused by PCR processes.

### Antibody modeling and molecular dynamics simulations

To generate a 3D structure for the immune complex of STn bound to the antibody, we used the VH/VL sequences to create a homology model for the antibody variable fragment (Fv), then refined the 3D structure by subjecting it to molecular dynamics simulations. Two homology models were built using PIGS server (http://circe.med.uniroma1.it/pigs), this tool is fast and available online. Three additional models were obtained using the recently developed knowledge-based AbPredict algorithm. AbPredict tool combines segments from various antibodies, then samples large conformations space resulting with the low energy homology models^26^.

**Figure 1:**
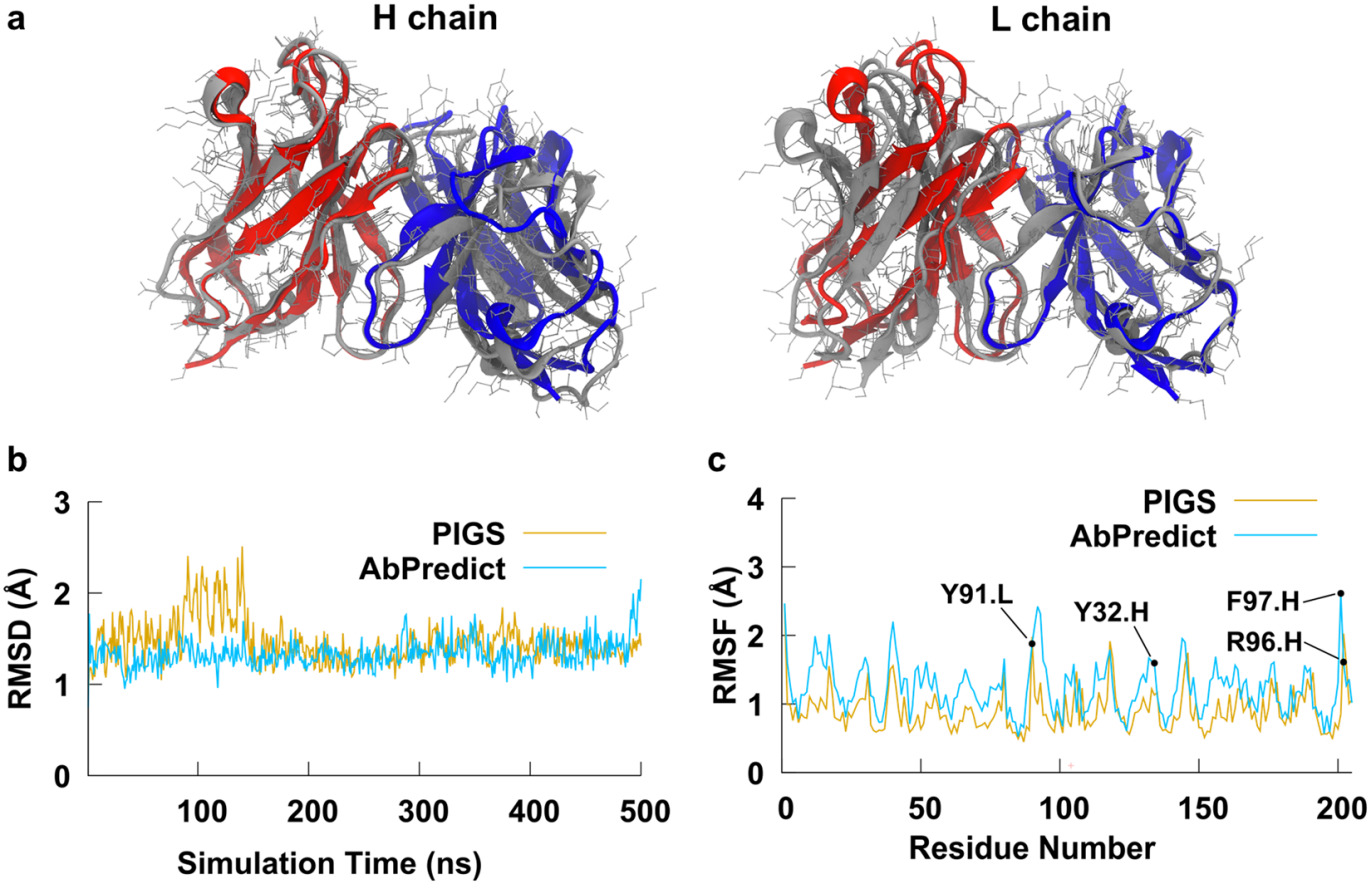
Structural modeling of TKH2 monoclonal antibody. (**a**) Structure-based alignment of TKH2 mAb models with the heavy and light chains of PIGS model shown in red and blue respectively, and the AbPredict model shown in grey. The leftmost alignment is of the heavy chain (111 atom pairs, 0.8 Å RMSD), and the rightmost alignment is with the light chain (103 atom pairs, 0.7 Å RMSD). The overall RMSD of 1.57 Å indicates a difference in the heavy and light chain relative position between the two models. (**b**) An RMSD plot of both structure models Cα atoms relative to their initial structures over the 500 ns MD simulation. (**c**) Average atomic fluctuations of Cα atoms over the 500 ns simulations of PIGS and AbPredict structure models without the ligand. Residues that were selected to be flexible during one of the docking protocols are labelled (VH−Y32, VH−R96, VH−F97 and VL−W91).

The fold of the protein backbone was very similar between the PIGS and AbDesign models (Cα Root Mean Square Deviation, RMSD, of 1.57 Å), however the sidechains differed more significantly (RMSD 2.35 Å) (Fig. 1a). We selected one model from each program for further analysis. PDB template entries sequences that were used to build the PIGS homology model had 89.9% (VL) and 82.46% (VH) identity to the TKH2 sequences, while in AbPredict model the identity to the TKH2 sequences were 91.9% for VL, 58.8% for L3, 86.0% for VH and 66.7% for H3. Both homology models were subjected to a 500 ns MD simulation in explicit water. An RMSD relative to the initial homology model showed that the model generated by PIGS was stable throughout the production simulation, whereas the Cα atom positions of the model from AbPredict were initially relatively more volatile, but stabilized after 30 ns of simulation (Fig. 1b). An RMSF (Root Mean Square Fluctuation) of the protein Cα atoms (Fig. 1c) revealed a similarity in the per-residue flexibility between both structures. The AbPredict model had higher average values in general due to the higher degree of mobility in the initial 30 ns of simulation (Fig. 1c), but overall both structures displayed a marked similarity in per-residue mobility. The degree of fluctuation was comparable to that observed during an MD simulation of a mAb crystal structure (data not shown).

### Computational docking and assessment of pose stability

We next generated a preliminary 3D model for the STn glycan ligand and used computational docking to generate putative poses of the STn ligand in the TKH2 combining site and subsequently performed molecular dynamics simulations to assess pose stability. 3D structures for the six known rotamers of Neu5Acα2–6GalNAcαOMe were generated using the GLYCAM-Web (www.glycam.org/cb)^27^ server that generates 3D models of carbohydrates based on the GLYCAM06 forcefield^28^. In the first protocol, each rotamer was rigidly docked to 25 snapshots from the MD simulation. The sidechains of four amino acids residues within the CDRs (VH−Y32, VH−R96, VH−F97 and VL−W91), that showed significant motion during the unliganded MD simulations (Fig. 1), were allowed to be flexible during the ligand docking. Twenty poses were generated for each docking run, resulting in 3,000 poses (=6 rotamers × 25 snapshots × 20 poses) for each model (PIGS and AbPredict). Out of the 6,000 screened poses, the resulting poses for each rotamer were clustered amongst themselves using a 2 Å cut-off (see Methods section for details) to remove similar poses. The top scoring pose from the top 20 clusters from each of the 6 rotamers (120 structures per homology model) were selected for MD simulation.

In a second protocol, the lowest energy rotamer of the glycan was flexibly docked to 150 snapshots from the MD simulation of the antibody model, generating additional 3,000 poses (20 poses retained from docking to 150 snapshots) for each homology model. Thus, a total of additional 6,000 poses were screened. After clustering, the 50 top scoring poses of each homology model were selected for MD simulation.

**Figure 2:**
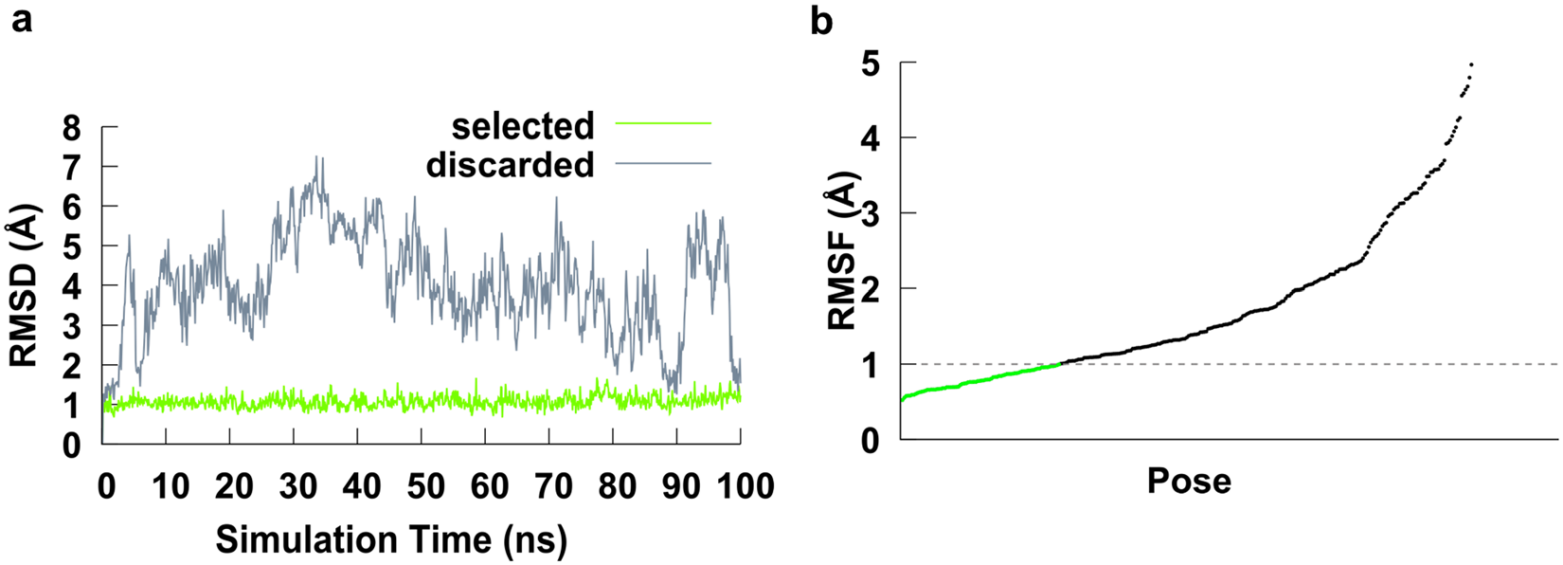
Pose filtering with RMSD/RMSF. (**a**) An example of a discarded pose (grey) that was repositioning in the binding site, and thus showed a high and variable RMSD, as well as a selected, stable pose (green). (**b**) All poses ordered by average RMSF during the last 10 ns of MD. Only poses that displayed a stable RMSD and therefore low RMSF for the last 10 ns of the simulation were selected. Poses that were not bound tightly by the protein (RMSF higher than 1.0 Å) were discarded.

In total, we screened 12,000 plausible models that were then narrowed down to 340 putative structures (120 from rigid protocol and 50 from flexible protocol for each homology model). These were then subjected to a 100 ns MD simulation to assess their stability in the antibody binding site. An RMSF analysis was performed on the last 10 ns of the simulation for each ligand. Poses that had a high degree of instability in the last 10 ns were filtered out (Fig. 2b), removing 260 poses while retaining 80 putative poses for further analysis.

### Experimental antibody specificity and glycan array alanine scanning

**Figure 3:**
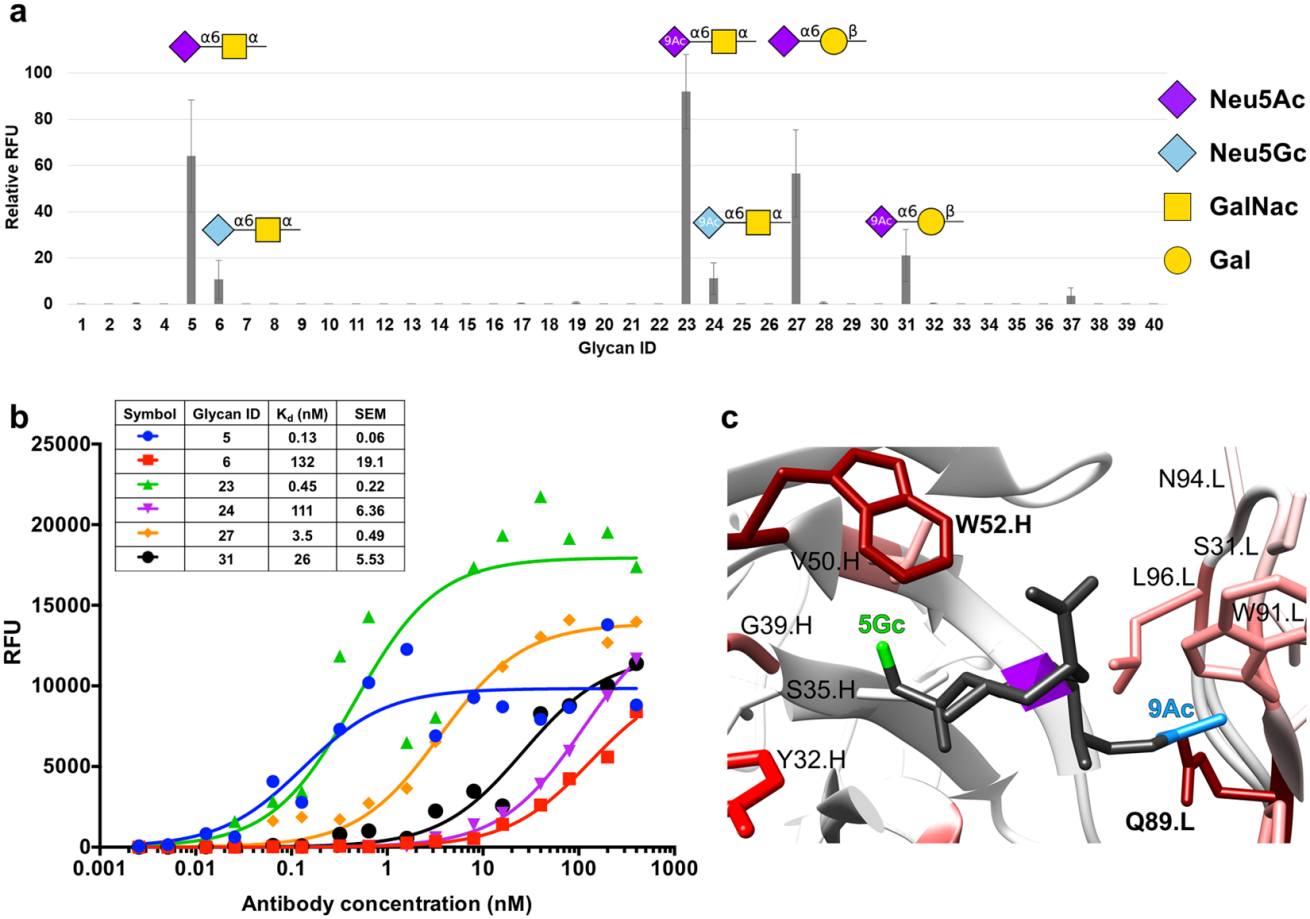
TKH2 glycan microarray analysis of specificity and affinity. (**a**) Binding of mTKH2 to diverse glycans was examined at 30, 12, 6 and 2.4 ng/µl by a sialoglycan microarray printed at four replicate spots per glycan. Relative fluorescence units (RFU) was calculated as percentage of maximal binding at each concentration, followed by averaging the relative RFU rank of the four tested antibody concentrations for each glycan (mean ± SEM; 9Ac denotes 9-*O*-acetylation on the sialic acids). Representative of two independent experiments (full data in supplementary Excel file). (**b**) Binding of the chimeric hTKH2 was tested at 16 serial dilutions (400–0.00256 nM) for the top six bound glycans (full data in supplementary Excel file). Apparent K_D_ was calculated according to non-linear fit with one-site specific binding using GraphPad Prism 6.0. (**c**) Modeling of the STn ligand within the antibody binding site. The *N*-glycolyl (5Gc; green) and 9-*O*-acetylation (9Ac; cyan) sialic acid derivatives were built onto the final 3D structure model of TKH2 (grey). Proximal amino acids in the antibody binding site are colored.

To further discriminate between models, and to assess the binding mechanism and specificity, the original mouse TKH2 antibody (mTKH2) and the human IgG Fc chimeric antibody (hTKH2) were purified from BM-4 and transfected 293A cells, respectively (Supplementary Fig. 1a–c). Antibody specificity was determined by screening the mAbs against a sialoglycan microarray^29,30^ (Fig. 3a; Supplementary Fig. 1d). Apparent binding curves were generated by titrating the antibody at multiple concentrations (16 serial dilutions, 400–0.00256 nM), and apparent K_D_ values derived by fitting an equilibrium binding model to the data (Fig. 3b). The measurement of apparent K_D_ values provides an important quantification of relative affinities^31^, which may otherwise only be qualitatively inferred from microarray data. The top binders were STn containing a terminal Neu5Ac (AcSTn; glycan #5; Neu5Acα2–6GalNAcα) or its 9-*O*-acetylated analog (glycan #23; 9Ac-AcSTn; 9-*O*-Ac-Neu5Acα2–6 GalNAcα). Also recognized were glycans #27 and #31 (Neu5Acα2–6Galβ and 9-*O*-Ac-Neu5Acα2–6 Galβ, respectively), which were similar to glycans #5 and #23, but differ by having a C2-hydroxyl group instead of an acetamido group in the penultimate sugar and a β-glycosyl linkage to the aglycon linker. Unlike other anti-STn antibodies^15^, TKH2 has a pronounced preference for AcSTn over GcSTn (STn with a terminal Neu5Gc), as demonstrated by the much lower affinity against glycans #6 (Neu5Gcα2–6GalNAcα) and #24 (9-*O*-Ac-Neu5Gcα2–6GalNAcα; 9Ac–GcSTn) (Fig. 3a,b). Figure 3c demonstrates the recognition of glycans #23 and #24 using a 3D structure with 9Ac–GcSTn. Galβ residue, found in glycan #27 and #31 is more relevant to *N-*glycosylation, that are not supposed to be recognized by TKH2, which was generated against the *O*-glycan target STn. It is possible that the high affinity together with the ligand high density on the glycan microarray led to this detection^29^. No binding was detected to any Neu5Acα2–3-containing glycan structures.

**Figure 4:**
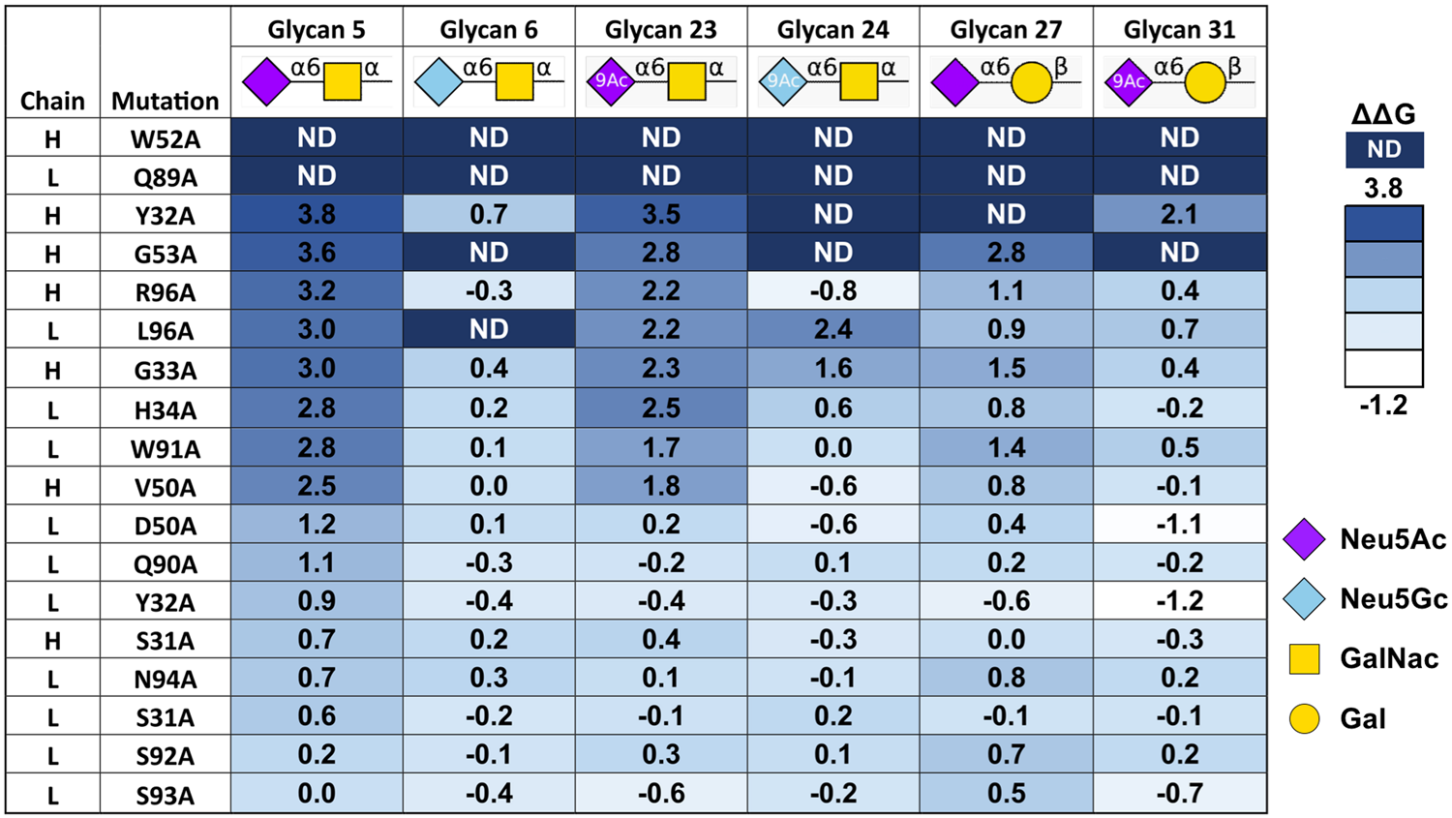
ΔΔG of apparent K_D_ fold change of alanine-scanning mutants. Apparent K_D_ [nM] of hTKH2 and related mutants were calculated as described in methods section. Then, the fold change for each glycan was calculated as apparent K_D_ of mutant Ab divided by the apparent K_D_ of the original Ab (relative apparent K_D_). The relative binding free energy (ΔΔG in kcal/mol) was then calculated [ΔΔG = RTln (apparent K_D_/reference apparent K_D_)]. ΔΔG serves to quantify the effect of the specific amino acid residue mutation to alanine on Ab reactivity: ΔΔG ≅ 0, ΔΔG > 0, ΔΔG < 0, respectively suggest that the mutation has minimal effect, destabilizing effect, or stabilizing effect on Ab binding (9Ac denotes 9-*O*-acetylation on the sialic acids).

Based on antibody structure models and CDR identification, 18 residues were selected for alanine scanning to determine their relative contribution to binding (16 residues within CDR regions). These mutated human-IgG chimeric antibodies were purified and their binding preferences were analyzed by the glycan microarray. Each purified antibody was applied at 16 different concentrations to generate a binding curve and calculate the apparent K_D_ against each glycan on the array (Supplementary Fig. 2). To evaluate the effect of each mutation on reactivity of the antibodies, the apparent K_D_ values for the mutants were converted to free energies and reported as the change in free energy (ΔΔG) relative to the wild-type antibody (Fig. 4). This analysis revealed that some mutations, such as VH–S31A and VL–S92A made only minor influences on the apparent K_D_ (ΔΔG close to 0) while others, such as VL–W91A and VH–G33A impaired the binding against all glycans, but to a greater extent for the glycans containing GalNAc instead of Gal (glycan #5 versus glycan #27; glycan #23 versus glycan #31). The latter observation suggests that these residues interact with the acetamido group of the GalNAc. The impact of some mutations, such as VH–W52A and VL–Q89A were so dramatic that it was not possible to calculate the apparent K_D_ for any glycan, suggesting they played key roles in forming binding site structure or affinity interactions with the ligand.

### Pose filtering with glycan STD-NMR and antibody alanine scanning

**Figure 5:**
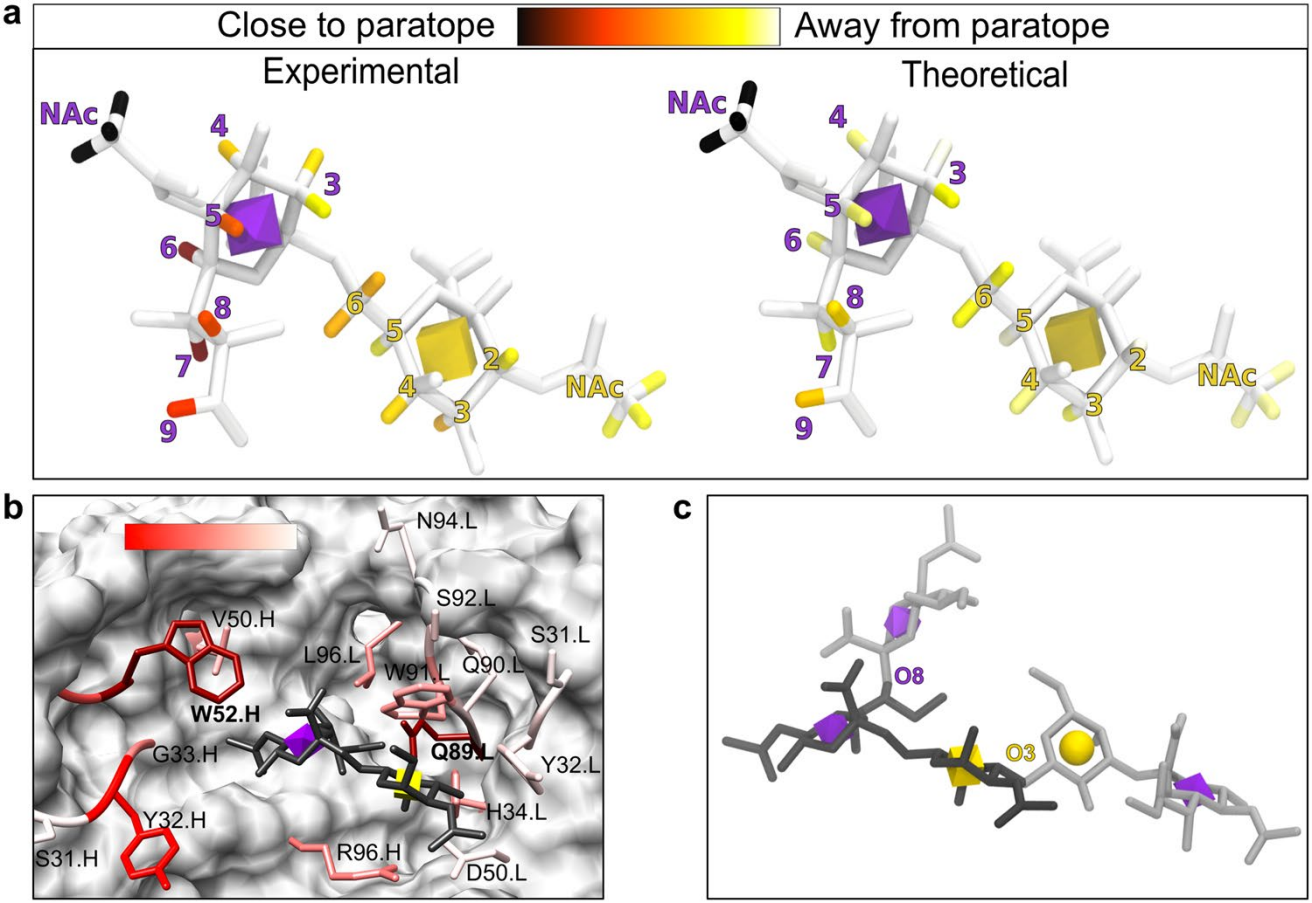
Experimental and computational cross validation of ligand-Ab complex. (**a**) Graphical representation of normalized STD-NMR for STn antigen in complex with TKH2. The experimental STD-NMR values mapped onto a 3D structure of the antigen (Neu5Acα2–6GalNAcα-R). The antigen is shown in white licorice, with signal intensity for the hydrogens shown on a color scale (increasing signal intensity from yellow to red to black; atoms with no signal are colored white). A 3D-SNFG icon^36^ in the center of the residue ring depicts the Neu5Ac (purple octahedron) and Gal (yellow cube) residue. The figure was generated using VMD 1.9.3^51^. (**b**) TKH2 binding site with residues experimentally mutated to alanine (licorice with a transparent surface). The residues are colored (white to red with a 2.2 gamma correction) by the change in apparent K_D_ values (array data) for Neu5Acα2–6GalNAcα-ProNH_2_ binding when that residue was mutated to an alanine. Mutation of residues VL–Q89A and VH–W52A (burgundy) resulted in undetectable binding. The 3D structure shown is the final structure from the 100 ns MD simulation of the selected pose. (**c**) The two branch points (light grey licorice) from Neu5Acα2–6GalNAcα (dark grey licorice) found within the known human glycome are exemplified by glycan 9187 (glycomeDB ID). A glycan with extensions at either the Neu5Ac O8 or the GalNAc O3 are not predicted to fit in the TKH2 combining site based on the generated complex model. Glycans containing Neu5Acα2–6GalNAcα with extensions at the O4 of either the Neu5Ac or GalNAc have not been observed in humans.

Saturation transfer difference NMR experiments determine the difference in nuclear Overhauser enhancement (nOe) magnetization transfer from the irradiated protein to a bound ligand then subsequently to a released ligand^32^. The relative increase in a ligand proton signal is proportional to its proximity to protons in the protein. Such data can provide valuable information related to the relative orientation of the bound glycan within the mAb combining site^33–35^. It is possible to compare the experimental STD enhancements with those computed for a docked pose, and filter out any poses whose theoretical STD enhancements are not in agreement with the experimental values^36^ (Table 1). The STD-NMR measurements mapped onto a 3D structure of the antigen Neu5Acα2–6GalNAcαOMe and the agreement between the predicted and experimental STDs is shown in Fig. 5a. Both the STD-NMR data and the theoretical structure (Fig. 5a,b; Supplementary Fig. 3; Supplementary Table 2) suggest a key role for the acetamido moiety in the binding of the antigen to TKH2, with that of the Neu5Ac residue being the most significant. The pose that gave the best agreement with the STD-NMR data was the most stable in the binding site during the last 10 ns of MD and resulted in a strong theoretical affinity. This pose, optimally selected out of the remaining 80 putative poses, was further validated by the experimental alanine scanning, as it placed the antigen proximal to key antibody residues (e.g. VL–Q89 and VH–W52 whose mutation to alanine resulted in undetectable binding; Fig. 4). In the selected unique pose (Fig. 5c) the sialic acid residue is proximal to both VH–W52 and VH–Y32, although it is not forming direct interactions with VH–Y32. The interaction with VH–W52 would be through the methyl group of Neu5Ac. The glycerol arm of sialic acid is positioned to interact directly with both VL–L96 and VL–Q89. Given the importance of the methyl and glycerol shown in the STD-NMR results, the proximity analysis can explain why the mutagenesis of VH–W52 and VL–Q89 to alanine abolished the binding. The GalNAc ring forms a stacking interaction with VL–W91 that is characteristic of carbohydrate binding^37^. The reducing terminal of the GalNAc is positioned such that glycans with either α or β anomeric configurations would be physically tolerated in the combining site.

**Table 1:** Docking poses that pass the docking rescore, RMSF and comparison to STD-NMR values filtering steps.

Pose ID	Docking rescore^a^	STD-NMR Correlation^a^	RMSF (Å)^a^
AbPredict.F.115.6	−9.5	0.74	0.6
PIGS.F.107.2	−7.9	0.68	0.8
PIGS.F.65.5	−8.1	0.63	0.9

^a^All calculations were performed on the last 10 ns of the MD simulation.

### Glycan array screening data support antibody-ligand model

The data from glycan array screening provided further experimental evidence for the importance of the acetamido moiety on Neu5Ac within the STn ligand when bound by TKH2 (Fig. 3a,b). This analysis clearly showed the reduced binding when the terminal sialic acid was rather Neu5Gc instead of Neu5Ac (Figs 3a,b and 4; Glycan IDs #6 and 24). The *N*-glycolyl moiety of Neu5Gc had a destabilizing effect perceived through reduced binding and a 1000-fold decreased affinity (Fig. 3b; Glycan IDs #5 and #6 are 0.13 nM to 132 nM, respectively, hence reduced affinity by at least 2 orders of magnitude). In contrast, the 9-*O*-acetylation sialic acid derivatives had a rather stabilizing effect (Figs 3a,b and 4; Glycan IDs #23 and 27), and were affected similarly to the original STn antigen Glycan ID #5 by the alanine scanning (Fig. 4). While glycan arrays currently hold a limited set of glycans that can be synthesized or properly purified, recent advances provide computational exploration of the much greater glycome for a more complete view on expected antibody specificity. Based on an examination of the optimal pose of STn in the binding site, the lack of measurable affinity for glycans terminating in Neu5Acα2–3 Gal sequences appears to arise from a loss of favorable interactions, rather than from unfavorable steric interactions. A low energy shape (-gauche φ angle)^38^ of the Neu5Acα2–3 Gal sequence could readily fit into the combining site when the Neu5Ac ring was superimposed onto that in the STn ligand (Supplementary Fig. 4). However, the Gal residue in the Neu5Acα2–3 Gal sequence was not located in the same position as in the STn ligand, and was unable to form the same stabilizing interactions with the antibody.

### Computational carbohydrate grafting for predicting putative off-targets within the known human glycome

The Gly-Spec webtool^39^ available at www.glycam.org/gr was used to perform computational carbohydrate grafting^40^ on the validated 3D model of the glycan-mAb complex with the known human sialyl-Tn-glycome. The technique first searches for human glycans that contain the antigen as part of their structure and then builds the 3D structure of that human glycan into the mAb binding site by growing out the larger glycan from the antigen “core” and adjusting the glycosidic linkages within normal ranges to relieve any steric overlaps. Glycans that can fit in the binding site without significant steric overlaps are putative off-targets for the specific antibody, and information such as disease/healthy state and tissue location can be retrieved from www.glycome-db.org using the glycan ID number. In the case of TKH2, 86 human glycans were found to contain the antigen within their structure, however none were predicted to be bound by TKH2. The orientation of the antigen in the TKH2 binding site occluded the glycerol arm of the sialic acid, as well as the O3 and O4 of the GalNAc (Fig. 5c). No larger glycans were found that displayed the antigen at the non-reducing terminal. Thus, the TKH2 Ab was predicted to be highly specific for Neu5Acα2–6GalNAcα- within the human sialyl-Tn-glycome. The computational carbohydrate grafting method is useful for predicting specificity for glycans that contain a high affinity binding determinant, however it does not directly address whether other binding determinants could bind with high affinity. Thus, the array data (Fig. 3a) was important to demonstrate that glycans with a Siaα2–3-linkage are generally excluded from TKH2 recognition. The repertoire of those experimentally recognized sialoglycans on the array further support the glycome-DB computational screening of the TKH2, demonstrating its high specificity for Neu5Acα2–6GalNAcα-R.

## Discussion

Important advances in antibody modelling and antigen docking have been made recently^35^. Yet the accuracy of computational docking alone is sensitive to a plethora of features and highly variable, thus the selection of the optimal theoretical antigen-antibody docking pose needs to be reinforced by experimental data^22,41,42^. Glycan microarray studies present the binding preferences of glycan-binding proteins with high resolution and can be used to guide MD simulation toward potent antibody-glycan interaction^40^. Modeling can further shed light on binding preferences determined in these arrays e.g. the effects of different linkers on binding^43^. Here we describe an experimental-computational approach to delineate the binding features of anti-carbohydrate antibodies, combining glycan microarray, site-directed mutagenesis, STD-NMR, modeling, docking and MD simulations. This method allowed to select one antibody-antigen model out of 12,000 plausible poses. The selected pose could be tightly associated to both the theoretical and experimental data, thereby supporting the wider implementation and applicability of this method to circumvent challenging co-crystalizing efforts.

STn is a tumor-associated carbohydrate antigen and antibodies against it are potential cancer theranostics^44^, which prompted efforts to target it by therapeutic cancer vaccines^13^. Several monoclonal antibodies against STn^15^ have been developed but none have been crystallized thus far. We show that the anti-STn TKH2 mAb is highly specific to a limited set of closely related glycans, and has exceptionally high affinity with apparent K_D_ in the nM range, as opposed to other anti-carbohydrate antibodies^9^. We used experimental STD-NMR to define STn glycan regions that are proximal to the TKH2 mAb surface, and site-directed mutagenesis to identify key-residues in the CDRs that are critical for antigen binding^33,40^. Additional antigen-antibody complexes could then be generated for related glycan antigens, by grafting glycan sub-structural features onto the optimal pose obtained by ligand docking. Subsequently, computational grafting^33,40^ provided an additional mechanism for pose validation, by enabling a direct comparison of the theoretical specificities for a range of glycans against the specificities observed by glycan array screening. An ideal model should be consistent with the STD-NMR, site-directed mutagenesis data, and glycan array data. With such a validated model, it was then possible to interpret the observed specificities in terms of molecular structure, and to formulate hypotheses regarding potential untested cross-reactivities. The Gly-Spec Webtool (www.glycam.org/gr)^39^ was developed to facilitate such specificity prediction^33^. The combined experimental and computational approach presented here provided a 3D structural basis for explaining the specificity of the anti-cancer mAb TKH2. The methods employed in this study are generally applicable to carbohydrate-mAb complexes, and therefore should be broadly useful for defining the structural origin of the specificity of anti-carbohydrate antibodies. Given the importance of carbohydrates as disease markers, and the challenges in characterizing carbohydrate-antibody complexes crystallographically, the ability to routinely generate experimentally-consistent 3D structures for carbohydrate-antibody complexes has the potential to significantly advance the development of therapeutic and diagnostic antibodies.

## Methods

### Cell culture

The BM-4 mouse hybridoma cells expressing TKH2 antibodies were obtained from the American Type Culture collection (ATCC). For culturing BM-4, cells were grown in RPMI-1640 medium supplemented with 10% heat inactivated fetal bovine serum (FBS), 2 mM L-glutamine, 100 units/ml penicillin and 0.1 mg/ml streptomycin, all from biological industries. For antibody purification, BM-4 cells were grown in DCCM-1 (biological industries) medium supplemented with 2 mM L-glutamine, 100 units/ml penicillin and 0.1 mg/ml streptomycin. The 293A human embryonic kidney cells were obtained from Life technologies. 293A cells were grown in DMEM (biological industries) supplemented with 10% heat inactivated fetal bovine serum (FBS), 2 mM L-glutamine, 100 units/ml penicillin and 0.1 mg/ml streptomycin. For antibody purification, 293A cells were grown in a medium contained equal amounts of RPMI-1640 and DMEM supplemented with 75 units/ml penicillin, 0.075 mg/ml streptomycin, 2 mM L-glutamine, 1% Nutridoma (Roche) and 1 mM sodium pyruvate (biological industries).

### Antibody purification

Wild type and mutated antibodies were purified from Hybridoma and 293A supernatants using protein A or protein G (GE healthcare). Antibodies concentrations were determined by BCA assays (Pierce), from 1 L hybridoma culture, ~20 mg mTKH2 antibodies were purified at a concentration of 3 mg/ml. From 25 ml culture of 293A cells transfected with hTKH2 plasmids, ~100 µg hTKH2 antibodies were purified at a concentration of 1.07 mg/ml.

### cDNA synthesis

RNA was extracted from the hybridoma cells BM-4 (ATCC) using TRIzol reagent according to the manufacturer instructions (Life) and then reverse transcribed to cDNA by qScript cDNA Synthesis Kit according to the manufacturer instructions (Quanta Biosciences).

### PCR amplification of anti-AcSTn variable regions

Primers were synthesized according to Tiller *et al*. 2009 (IDT Syntezza). First PCR round was performed using Q5 high fidelity DNA polymerase (New England BioLabs), with 0.728 ng cDNA in 4 μl as a template (equals to cDNA from 2.4 cells), 300 μM each dNTP, 1.2 U Q5 hot start high fidelity DNA polymerase, 200 nM each primer (Supplementary Table 1 primers #1–14) The first round was performed at 94 °C for 15 minutes followed by 50 cycles of 94 °C for 30 seconds (s), 55 °C for 30 s, 72 °C for 55 s and final incubation of 72 °C for 10 minutes. Amplified PCR products were extracted from gel using Wizard SV GEL & PCR clean-up system (Promega) according to manufacturer instructions. For VH product, a second PCR round was performed. Reaction was made in Q5 reaction buffer, with 2 μl of first PCR round product as the template, 300 μM each dNTP, 1.2U Q5 hot start high fidelity DNA polymerase, 200 nM each primer (Supplementary Table 3a primers #1 and #15). Reaction conditions of this round were 94 °C for 15 minutes followed by 50 cycles of 94 °C for 30 s, 65 °C for 30 s, 72 °C for 45 s and final incubation of 72 °C for 10 minutes. Amplified PCR products were extracted from gel. Third PCR round was performed for the amplified VH product. The reaction was carried out in Q5 reaction buffer, with 2 μl of second PCR round product as the template, 300 μM each dNTP, 1.2U Q5 hot start high fidelity DNA polymerase, 200 nM each primer (Supplementary Table 3a primers #16 and #17). Reaction conditions of this round were 94 °C for 15 minutes followed by 50 cycles of 94 °C for 30 s, 62 °C for 30 s, 72 °C for 45 s and final incubation of 72 °C for 10 minutes. Amplified PCR products were extracted from gel. The VL PCR product was amplified in a second nested PCR. The reaction was carried out in Q5 reaction buffer, with 2 μl of first PCR round result as the template, 300 μM each dNTP, 1.2U Q5 hot start high fidelity DNA polymerase, 200 nM each primer (Supplementary Table 3a primers #18 and #19). Reaction conditions of this round were 94 °C for 15 minutes followed by 50 cycles of 94 °C for 30 s, 62 °C for 30 s, 72 °C for 45 s and final incubation of 72 °C for 10 minutes. Amplified PCR products were extracted from gel.

### Cloning VH/VL into expression vectors

Purified variable regions were first cloned into pGEM-T easy vector (Promega). First, dATPs were added to the insert edges using BIOTAQ DNA Polymerase (bioline) according to manufacturer’s instructions. Then, inserts with A-overhang were ligated to pGEM-T easy vector. DH5α cells were transformed with pGEM-T easy vector containing anti-AcSTn variable inserts. In order to purify the variable inserts, light chain plasmids were digested by AgeI-HF (New England BioLabs), and BsiWI (New England BioLabs), heavy chain plasmids were digested by *Age*I-HF (New England BioLabs) and *Sal*I-HF (New England BioLabs). Digested products were extracted from 1% agarose gel. Human IgG (hIgG) expression vectors (FJ475055, FJ475056) were kindly provided by Prof. Patrick Wilson (University of Chicago). Plasmids for heavy and kappa light chain were digested with restriction enzymes (heavy chain expression vector with *Age*I-HF and *Sal*I-HF, light chain expression vector with *Age*IF-HF and *BsiW*I). Vectors were then treated with Antarctic Phosphatase (New England BioLabs) in order to prevent self-closure of the vector. Inserts were ligated with hIgG expression vectors using T4 DNA Ligase (New England BioLabs) and plasmids were transformed into DH5α cells.

### Alanine scanning

Residues were mutated to Alanine by PCR. NEBaseChanger web tool (http://nebasechanger.neb.com/) was used to design the primers for the mutagenesis and the primers are shown in Supplementary Table 3b. PCR reaction was performed in Q5 reaction buffer (New England Biolabs B9027S) supplemented with GC enhancer, with 18 ng plasmid DNA as the template, 200 μM each dNTP, 1U Q5 hot start high fidelity DNA polymerase (New England Biolabs M0493L), 500 nM each primer (Supplementary Table 3b), in a total volume of 40 μl. The reaction was performed at 98 °C for 30 followed by 25 cycles of 98 °C for 10 s, 58–69 °C (depend on the primers Ta) for 20 s, 72 °C for 150 s and final incubation of 72 °C for 2 minutes. PCR products were digested with *Dpn*I restriction enzyme (New England BioLabs). The reaction was performed in cut smart buffer, with 10 μl of PCR products and 1 μl *Dpn*I enzyme (New England Biolabs) in a total volume of 50 μl. The reaction was performed in 37 °C for 8 hours. After digestion, 10 μl of the reaction was transformed into DH5α competent cells as described above. Colonies were picked, grown in LB-Amp media overnight, and plasmids were purified with NucleoSpin Plasmid EasyPure kit according to manufacturer instructions (Macherey-Nagel) Plasmids were sent to sequencing to validate the mutation.

### Transfection of 293A cells

293A cells were grown to 80–90% confluency and evenly spread out across the 150 mm × 25 mm tissue culture plate. A mixture of 2.4 ml of DMEM and 9 µg of each antibody expression vector were prepared. 100 μg of polyethylenimine (PEI; Polysciences) were added to the prepared DMEM and DNA mixture that was immediately vortexed, and incubated at RT for 15 minutes. All culture media but 18 ml was removed from the plate to be transfected. Then, 2.5 ml of the DMEM, DNA, PEI mixture were supplemented to the plate. Subsequent to rocking the plate to ensure even distribution, cells were incubated at 37 °C with 5% CO_2_ for 24 hours. Culture media was changed to basal media, 25 ml per plate for incubation of 4 days in 37 °C with 5% CO_2_. Supernatant was collected four days later^45^.

### Sialoglycan microarray fabrication

Arrays were fabricated with NanoPrint LM-60 Microarray Printer (Arrayit) on epoxide-derivatized slides (Corning 40044) with 16 sub-array blocks on each slide. Glycoconjugates were distributed into one 384-well source plates using 4 replicate wells per sample and 8 µl per well (Version 2.0). Each glycoconjugate was prepared at 100 µM in an optimized print buffer (300 mM phosphate buffer, pH 8.4). To monitor printing quality, replicate-wells of human IgG (80, 40, 20, 10, 5, 0.25 ng/µl in PBS + 10% glycerol) and AlexaFlour-555-Hydraside (Invitrogen A20501MP, at 1 ng/µl in 178 mM phosphate buffer, pH 5.5) were used for each printing run. The arrays were printed with four SMP3 pins (5 µm tip, 0.25 µl sample channel, ~100 µm spot diameter; Arrayit). Each block (sub-array) has 20 spots/row, 20 columns with spot to spot spacing of 275 µm. The humidity level in the arraying chamber was maintained at about 70% during printing. Printed slides were left on arrayer deck over-night, allowing humidity to drop to ambient levels (40–45%). Next, slides were packed, vacuum-sealed and stored at room temperature (RT) until used.

### Sialoglycan microarray binding assay

Slides were developed and analyzed as previously described^29^ with some modifications. Slides were rehydrated with dH_2_O and incubated for 30 min in a staining dish with 50 °C pre-warmed ethanolamine (0.05 M) in Tris-HCl (0.1 M, pH 9.0) to block the remaining reactive epoxy groups on the slide surface, then washed with 50 °C pre-warmed dH_2_O. Slides were centrifuged at 200 × g for three min then fitted with ProPlate™ Multi-Array 16-well slide module (Invitrogen) to divide into the sub-arrays (blocks). Slides were washed with PBST (0.1% Tween 20), aspirated and blocked with 200 µl/sub-array of blocking buffer (PBS/OVA, 1% w/v ovalbumin, in PBS, pH 7.3) for 1 hour at RT with gentle shaking. Next, the blocking solution was aspirated and 100 µl/block of 1:100 diluted sera or purified anti-Neu5Gc antibodies in 10 ng/µl diluted in PBS/OVA were incubated with gentle shaking for 2 hours at RT. Slides were washed three times with PBST, then with PBS for 2 min. Bound antibodies were detected by incubating with secondary detection diluted in PBS, 200 µl/block at RT for 1 hour, Cy3-anti Human IgG 1.2 µg/ml (Jackson Immunoresearch). Slides were washed three times with PBST then with PBS for 10 min followed by removal from ProPlate™ Multi-Array slide module and immediately dipping in a staining dish with dH_2_O for 10 min with shaking, then centrifuged at 200 × g for 3 min. Dry slides immediately scanned.

### Array slide processing

Processed slides were scanned and analyzed as described at 10 μm resolution with a Genepix 4000B microarray scanner (Molecular Devices) using 300 gain. Image analysis was carried out with Genepix Pro 6.0 analysis software (Molecular Devices). Spots were defined as circular features with a variable radius as determined by the Genepix scanning software. Local background subtraction was performed.

### Array apparent K_D_ calculations

Slides were developed with purified hTKH2 wild-type and mutant antibodies at serial dilutions ranging from 60–3.84 × 10^−4^ ng/µl (400–0.00256 nM) in blocking buffer (PBS/OVA). Apparent K_D_ was calculated according to non-linear fit with one-site specific binding using GraphPad Prism 6.0.

### Molecular Modeling

#### Modeling of TKH2 mAb

VH and VL models were built using either PIGS server (http://circe.med.uniroma1.it/pigs) or by the recently developed knowledge-based AbPredict algorithm^26^. AbPredict program models the antibody variable domain by assembling backbone fragments from natural antibodies and recombining them *in silico*; the sequence of the query antibody is threaded on these backbone models, relaxed using Rosetta, and three clustered low-energy conformations are chosen as the predicted structures^19^.

#### Modeling of antigen

GLYCAM-Web (www.glycam.org/cb)^27^ was used to generate 3D models Neu5Acα2–6GalNAcα-OMe. As the α2–6-sialyl linkage is known to adopt multiple rotamers in solution, it was represented using six separate 3D structures for each permutation of rotamer combination (φ ≈ −60° or 180°, φ: C1-C2-O6′-C6′, ω ≈ 60°, −60° or 180°, ω: O6-C6-C5-O5).

#### Molecular Dynamics Simulations of Homology Models

All MD simulations were performed using the Amber14 software suite. Using tleap^46^, the 3D structures were placed in a cubic box of TIP5P^47^ water with a 10 Å water buffer with counterions to neutralize the system. The FF14SB^48^ and Glycam06j^28^ force fields were employed for the protein and the carbohydrate respectively. Protein 1–4 scaling factors were used for the protein portion only. Cut-offs of 10.0 Å for vdWs and 8.0 Å for electrostatics were employed. Initial energy minimization (10,000 steps steepest decent followed by 10,000 steps conjugate gradient) was performed with Cartesian restraints (5 kcal/mol throughout all phases) on all solute heavy atoms to optimize the water molecules position and orientation. The same restraints were employed during a 400 ps nPT equilibration phase at 300 °K. This was followed by a 1 ns structural equilibration phase with Cartesian restraints on protein Cα. The atom positions and velocities from the last step of equilibration were used to start a 100 ns production run, with Cartesian restrains on the Cα atoms of the residue in heavy and light chain that connect to the larger Ab structure.

#### General Docking Protocol

All docking calculations were performed using “Vina-Carb 1.1.2”^21^, an extension to the Autodock Vina program^49^. The antibody CDR co-ordinates were transformed to the origin and aligned to the z-axis and an energy range of 10, with chi values of 1 and 2 were selected as described^21^. Twenty modes were output per docking run.

#### Rigid ligand docking to a flexible receptor

Twentyfive snapshots were extracted at regular intervals from the MD simulation using cpptraj. Each of the six rotamers of Neu5Acα2–6GalNAcα-OMe was docked rigidly to each snapshot of the antibody. Four protein residues (Y32.H, R96.H, F97.H and W91.L) whose sidechains were flexible in the MD were set as flexible during the docking.

#### Flexible ligand docking to a rigid receptor

150 snapshots were extracted at regular intervals from the MD simulation using cpptraj^50^. The lowest energy rotamer of Neu5Acα2–6GalNAcαOMe, as reported by GLYCAM-Web^39^, was docked with flexible exocyclic bonds to each snapshot of the receptor.

#### Clustering

For each glycan rotamer, the 500 poses generated by rigid docking were clustered with a density-based scan (DBSCAN) in cpptraj program (a program designed to load and analyze molecular dynamics trajectories and relevant data sets derived from their analysis; 2 Å cutoff, minpoints = 1 and no sieving). For each rotamer, the top scoring pose from the top 20 most populated clusters was subjected to MD simulation (120 poses in total).

#### Post Filtering of Docked Poses using Molecular Dynamics Simulations

The same protocol as for “*Molecular Dynamics Simulations of Homology Models”* was followed, except that the production run was 100 ns.

#### Rescoring using autodock

Snapshots were extracted at regular from the last 1 ns of the simulation using cpptraj, and rescored in place using Vina-Carb.

### STD-NMR

A 30 µL, 6.3 mM solution of Neu5Acα2–6GalNAcα1-propylamine in water was lyophilized and redissolved in 90 µL D_2_O to make a 2.1 mM solution of ligand. 30 µL of the stock solution of purified mTKH2 antibody in water (3.05 µg/µL or approximately 20 µM) was diluted to 90 µL with D_2_O. Reference 1D proton spectra were collected with these samples on an Agilent 600 MHz spectrometer equipped with a 3 mm cryoprobe using a standard Agilent ‘dpfgse-water’ pulse sequence for water suppression. The STD sample was prepared by mixing 45 µL of the 2.1 mM ligand solution and 45 µL of the 20 µM mAb stock solution to give an approximate 100:1 molar ratio (10 µM Ab, 1.03 mM ligand). Preliminary data was collected at 600 MHz with the Agilent ‘dpfgse-satxfer’ pulse program, with irradiation at –1.5 ppm for a range of times. The above STD sample was then lyophilized, redissolved in 99.96% D_2_O, and used to collect reference proton, and STD data at 900 MHz with 2 s irradiation time, which was used in the analysis. Data was processed using Mestrenova software (Mestrelab Research S.L.) with 1 Hz Gaussian line broadening, zero filling and 3^rd^ order polynomial baseline correction. Integral values were obtained by line fitting each multiplet and deconvoluting overlapped peaks when possible. Resonance assignments were based on literature values (www.casper.organ.su.se/casper) and confirmed with analysis of COSY, TOCSY, HSQC and HMBC data collected at 600 and 900 MHz using standard Agilent pulse sequences.

## Electronic supplementary material

Supplementary information

Supplementary data
