# Supplementary material for: A combined computational-experimental approach to define the structural origin of antibody recognition of sialyl-Tn, a tumor-associated carbohydrate antigen
Source: Sci Rep. 2018 Jul 17;8:10786. doi: 10.1038/s41598-018-29209-9 (PMC6050261; doi:10.1038/s41598-018-29209-9)
Supplement: Supplementary file 1 — Supplementary information [file 41598_2018_29209_MOESM1_ESM.docx]

*May 23, 18*

*SUPPLEMENTARY INFORMATION*

**A combined computational-experimental approach to define the structural origin of antibody recognition of sialyl-Tn, a tumor-associated carbohydrate antigen**

Ron Amon^1#^, Oliver C. Grant^2#^, Shani Leviatan Ben-Arye^1^, Spandana Makeneni^2^, Anita K. Nivedha^2^, Tal Marshanski^1^, Christoffer Norn^3^, Hai Yu^4^, John N. Glushka^2^, Sarel J. Fleishman^3^, Xi Chen^4^, Robert J. Woods^2#^ and Vered Padler-Karavani^1#^

^1^Department of Cell Research and Immunology, The George S. Wise Faculty of Life Sciences, Tel Aviv University, Tel Aviv 69978, Israel; ^2^Complex Carbohydrate Research Center, University of Georgia, Athens 30606, GA, USA; ^3^Department of Biomolecular Sciences, Weizmann Institute of Science, Rehovot 76100, Israel; ^4^Department of Chemistry, University of California-Davis, Davis, CA, USA

# Equal contribution

**Keywords:** Anti-carbohydrate antibodies, computational modeling, glycan microarray, tumor-associated carbohydrate antigen, sialic acid

**Running title:** **Structural origin of carbohydrate recognition by a monoclonal antibody**

**Authors to whom correspondence may be addressed:**

Vered Padler-Karavani: Tel: +972-3-640-6737. Fax: +972-3-642-2046. E-mail address: [vkaravani@post.tau.ac.il](mailto:vkaravani@post.tau.ac.il)

Robert J. Woods: Tel: +1-706-542-4454. Fax: +1-706-542-4412. E-mail address: rwoods@ccrc.uga.edu

**Conflict-of-interest:** The authors declare no financial and non-financial competing interests

**Figures**

**a.**

**
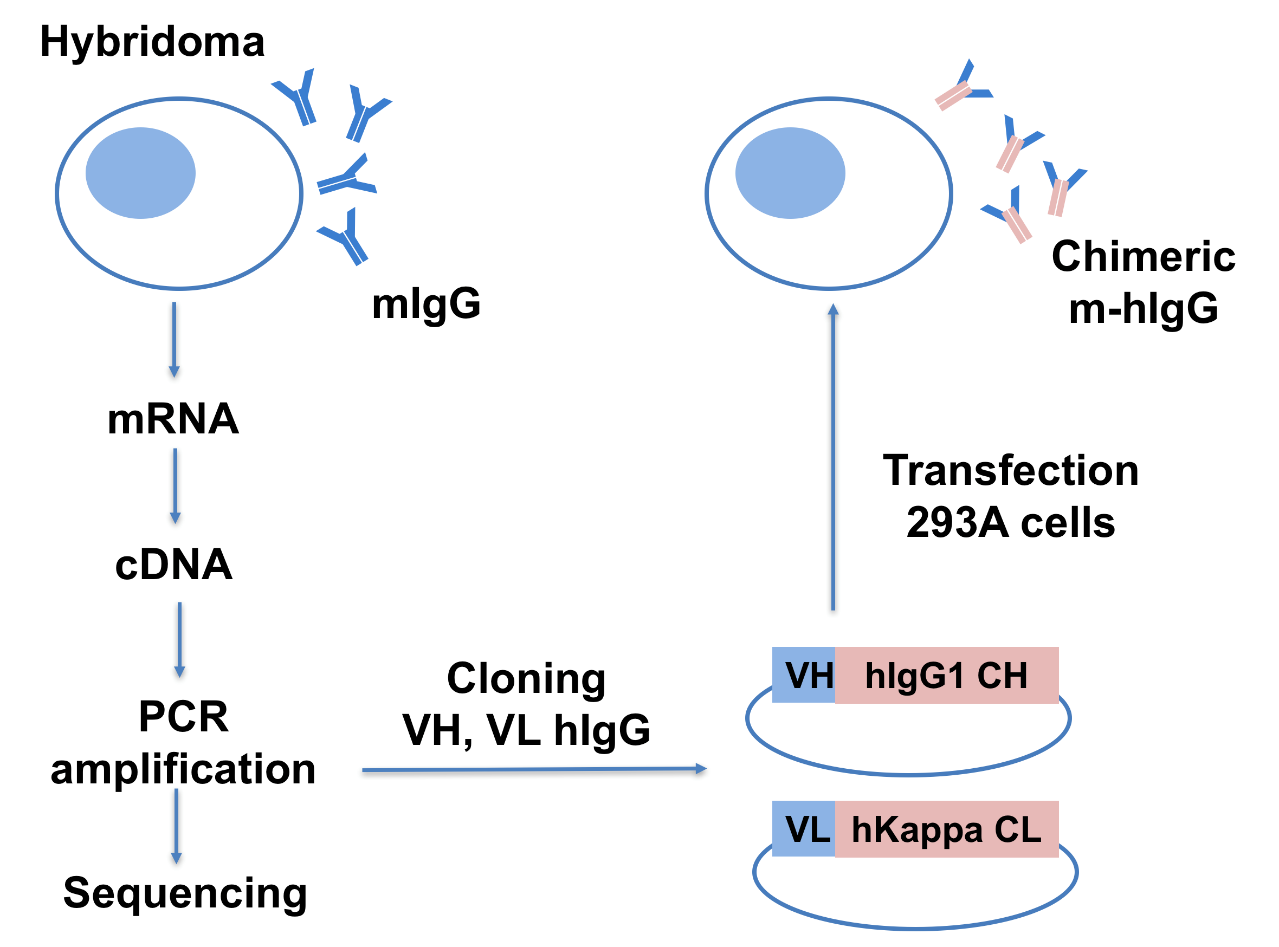
**

**b.**

**
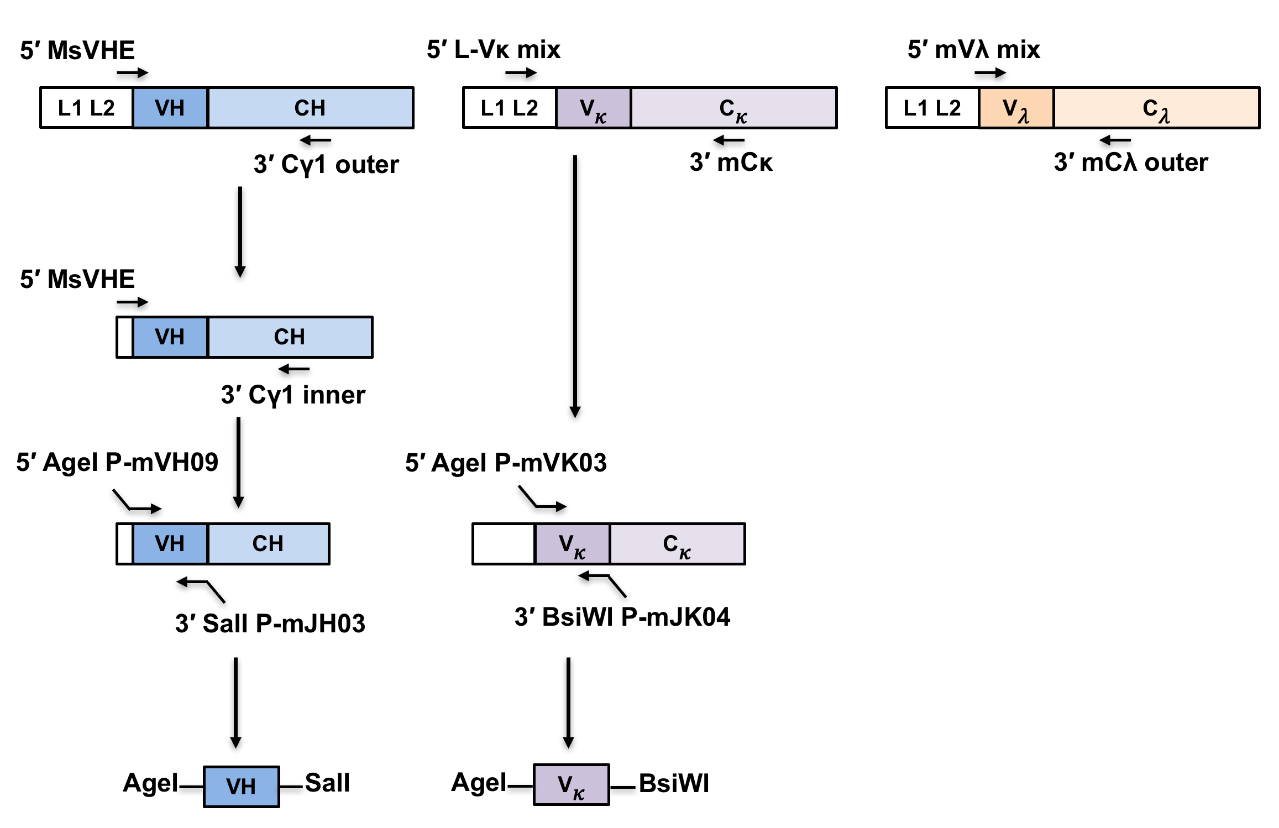
**

**c.**

**
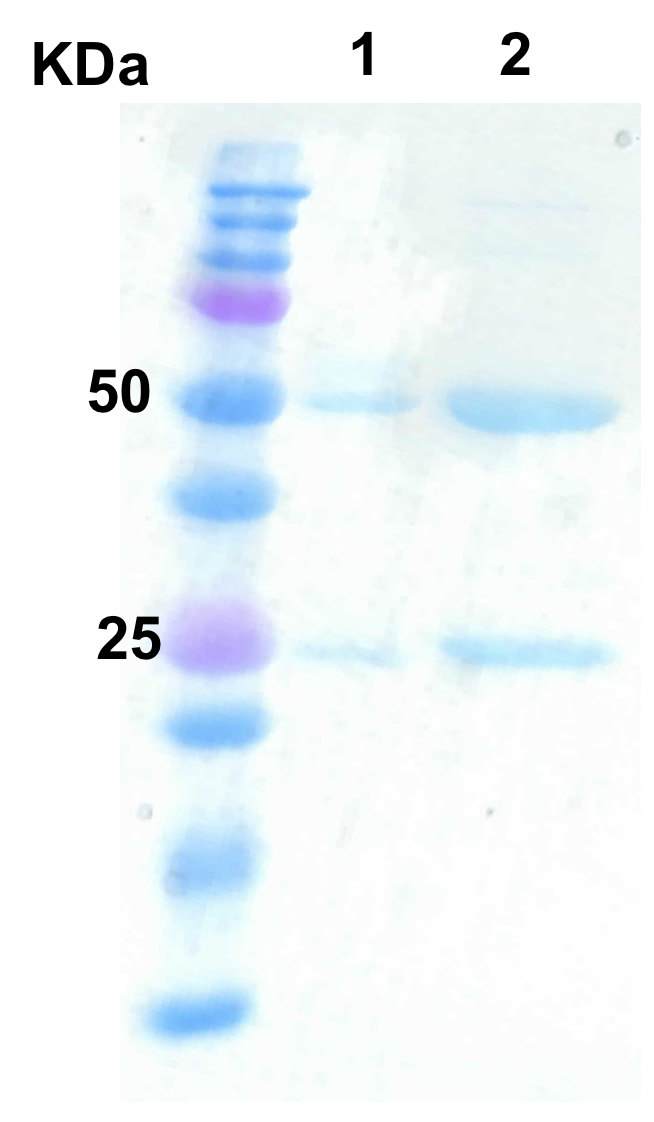
**

**d.**

**Supplementary Figure 1. Strategy for obtaining the VH and VL sequences of TKH2 mAb.** (**a**) Cloning and transfection process. (**b**) PCR amplification cycles diagram. (**c**) SDS-PAGE of mTKH2 and hTKH2. 5 µg of each of the purified antibodies was loaded onto 12.5% polyacrylamide gel, run for 20 minutes at 60 v then for 1 hour at 120 v. The gel was then stained with Bio-safe Coomassie (lane 1: mTKH2; lane 2: hTKH2). (**d**) Binding of hTKH2 to glycan microarray at 60 ng/µl (left) and 30 ng/µl (right).

**Supplementary Figure 2. Apparent K_D_ measurements of hTKH2 and its alanine mutants against glycan #5.** hTKH2 and its related alanine mutants were examined on sialoglycan microarrays at 16 serial concentrations (400-0.00256 nM). Apparent K_D_ was calculated for the hTKH2 top 6 reactive glycans according to non-linear fit with one-site specific binding using GraphPad Prism 6.0. Here we show an example of apparent K_D_ calculations of hTKH2 and its related alanine mutants against glycan #5 (K_D_ ± SEM). Mutants VL–Q89A and VH–W52A resulted in undetectable binding therefore apparent K_D_ could not be calculated.

**
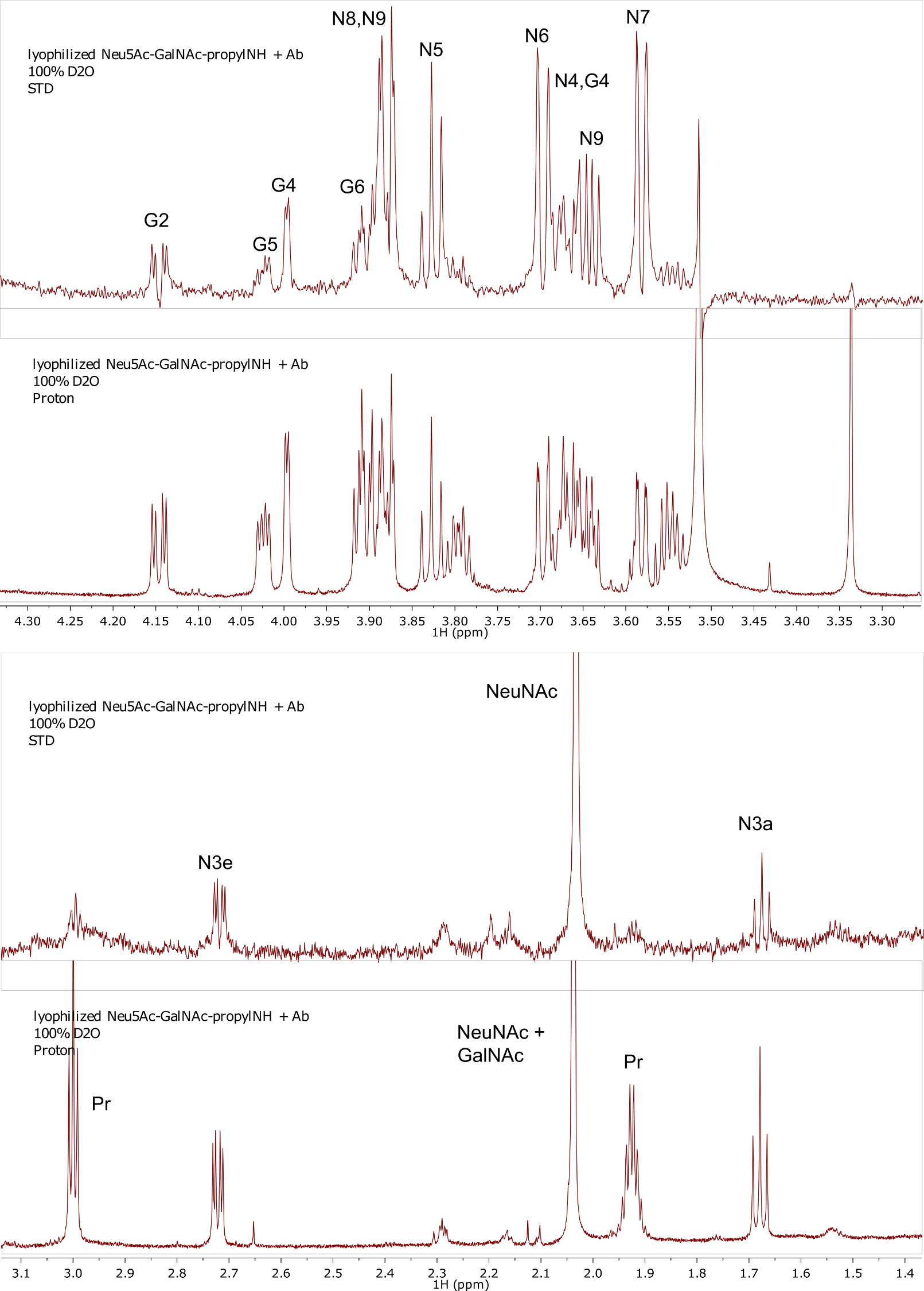
**

**Supplementary Figure 3. STD-NMR measurements of STn antigen binding to TKH2 antibody.** Lyophilized Ab mixed with Neu5Acα2-6GalNAcα1-propylamine in a ~100:1 ratio in D2O. The upper two panels compare regions of the STD and regular proton 900 MHz spectra, and the bottom panel shows a ^13^C-HSQC spectrum with resonance assignments (G = GalNAc, N = Neu5NAc, Pr = propylamine).  It is clear that overlap of the Neu5Ac-H4 and Neu5Ac-H6 peaks with GalNac-H6 can lead to ambiguous STD intensities.  However, the STD peaks for other Neu5Ac positions (e.g. N3,5,7,8,9) are clear and generally more intense than the GalNAc peaks. The GalNAc and Neu5NAc methyl signals at 2.04 ppm are distinct and were assigned using ^13^C-HMBC data (not shown) confirming the major STD interaction was with the Neu5NAc methyl group. Signals from the propylamine aglycon do not show much signal in the STD spectrum, which indicates that the observed STD peaks are real and not subtraction artefacts.

**
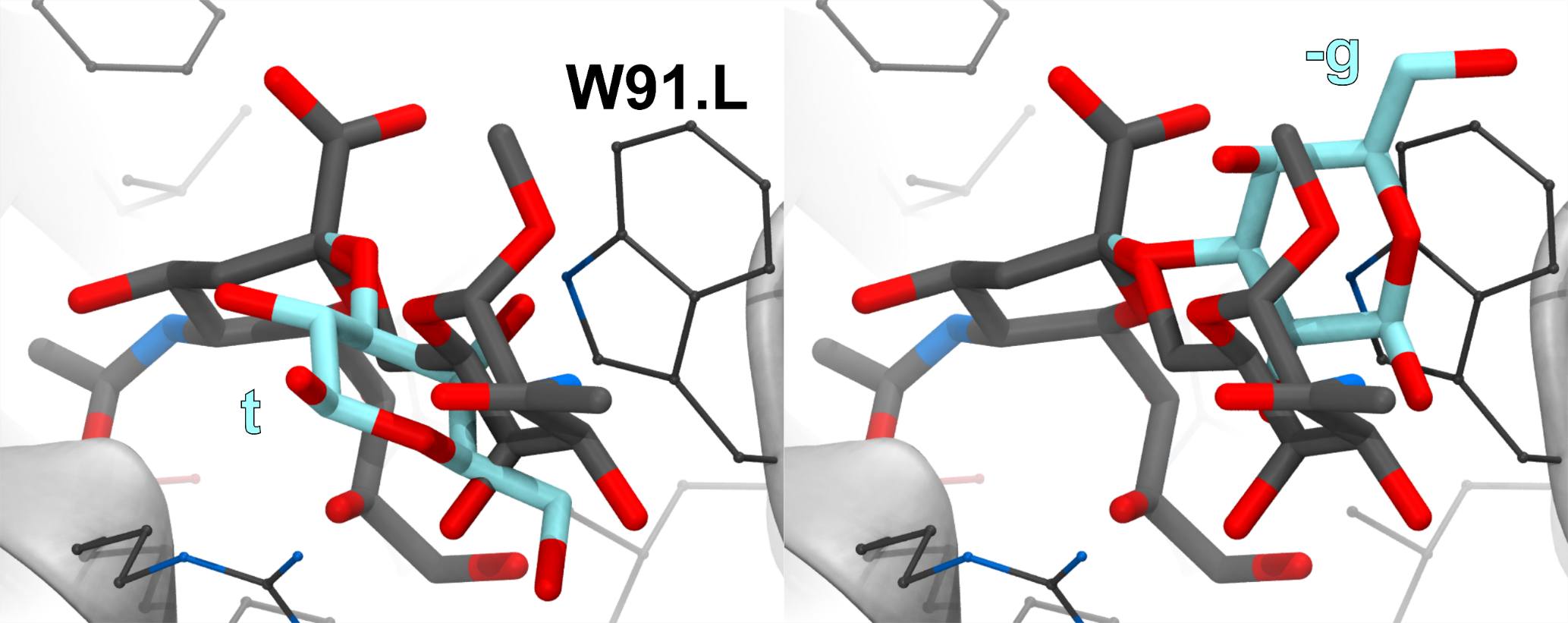
**

**Supplementary Figure 4. Neu5Acα2**–**3Gal antigen binding to TKH2 antibody.** The trans (180°, t, licorice with atom coloring and cyan carbons) and -gauche (-60°, -g, licorice with atom coloring and cyan carbons) conformers of Neu5Acα2-3Galβ were superimposed onto the sialic acid of the selected pose of Neu5Acα2-6GalNAcα (licorice with atom coloring and grey carbons) in the TKH2 combining site (ribbon and cpk). Neither shape can mimic the stacking interaction between W91.L and the 6-linked GalNAc. The t shape places Gal ring in a different position to, and with the opposite face towards, the W91.L than the 6-linked structure. The g shape also places the Gal ring in a different position, and where it would have steric overlaps with W91.L.

**Supplementary Table 1.** (**a**) DNA sequence of TKH2 variable heavy and light chains (VH and VL, respectively). (**b**) Amino acid sequence of variable heavy and light chains (VH and VL, respectively), including the variable regions according to KABAT numbering. (**c**) KABAT numbering of TKH2 amino acids divided to FR and CDRs.

**a.**

**Heavy chain variable DNA sequence:***

**G**AGGTGCAGCTG**C**AGGAGTCTGGACCTGGCCTGGTGGCGCCCTCACAGAGCCTGTCCATCACATGCACTGTCTCAGGGTTCTCATTAATCAGCTATGGTGTAAGCTGGGTTCGCCAGCCTCCAGGAAAGGGTCTGGAGTGGCTGGGAGTAATATGGGGTGACGGGAGCACAAATTATCATTCAACTCTCATATCCAGACTGAGCATCAACAAGGATAACTCCAAGAGCCAAGTTTTCTTAAAACTGAACAGTCTGCAAACTGATGACACAGCCACGTACTACTGTGTCGGACCCCGGTTTGCTTACTGGGGCCAAGGGACTCTGGTCACTGTCTCTGCA

* Sequence of variable heavy chain contains two mutations (red) derived from the primer used to amplify the segment. The first mutation changed the amino acid from glutamine to glutamate and the second mutation changed the amino acid from lysine to glutamine. To our knowledge, these two mutations did not affect the binding.

**Light chain variable DNA sequence:**

CAAATTGTTCTCACCCAGTCTCCAGCAATCATGTCTGCATCTCCAGGGGAGAAGGTCACCATGACCTGCAGTGCCAGCTCAAGTGTAAGTTACATGCACTGGTACCAGCAGAAGTCAGGCACCTCCCCCAAAAGATGGATTTATGACACATCCAAACTGACTTCTGGAGTCCCTGCTCGCTTCAGTGGCAGTGGGTCTGGGACCTCTTACTCTCTCACAATCAGCAGCATGGAGGCTGAAGATGCTGCCACTTATTACTGCCAGCAGTGGAGTAGTAATACGCTCACGTTCGGTGCTGGGACCAAGCTGGAGCTGAAA

**b.**

**Heavy chain variable amino acid sequence:**

🡨------------FR 2------------🡪

🡨---------------CDR 2-------

EVQLQESGPGLVAPSQSLSITCTVSGFSLISYGVSWVRQPPGKGLEWLGVIWGDGSTNYHSTLISRLSINKDNSKSQVFLKLNSLQTDDTATYYCVGPRFAYWGQGTLVTVSA

🡨-------FR 4---------🡪

🡨CDR 3🡪

🡨----------------------------------FR 3--------------------------------------🡪

----🡪

🡨----------------------------------FR 1--------------------------------🡪

🡨CDR 1🡪

**Light chain variable amino acid sequence:**

🡨---------------

🡨--CDR 2--🡪

🡨------------FR 2----------------🡪

🡨----CDR 1-------🡪

🡨---------------------------FR 1----------------------🡪

QIVLTQSPAIMSASPGEKVTMTCSASSSVSYMHWYQQKSGTSPKRWIYDTSKLTSGVPARFSGSGSGTSYSLTISSMEAEDAATYYCQQWSSNTLTFGAGTKLELK

🡨-------FR 4-------🡪

🡨-----CDR 3----🡪

-------------------FR 3-------------------------------------🡪

**c.**

| **Region** | **Heavy** | **Light** |
| --- | --- | --- |
| FR1 | 1-30 | 1-23 |
| CDR1 | 31-35 | 24-34 |
| FR2 | 36-49 | 35-49 |
| CDR2 | 50-65 | 50-56 |
| FR3 | 66-94 | 57-88 |
| CDR3 | 95-102 | 89-97 |
| FR4 | 103-113 | 98-107 |

**Supplementary Table 2.** **STD-NMR peak assignment of STn (Neu5Acα2**–**6GalNAcα1-propylamine) antigen binding to TKH2 antibody.**

| Peak assignment | No. of overlapped protons | Normalized integral values from linefitting peaks^a,b^ |
| --- | --- | --- |
| g2 | 1 | 22 |
| g4 | 1 | 27 |
| g5 | 1 | 18 |
| g6,g3 | 1 | 34 |
| n8,9 | 2 | 51 |
| n5 | 1 | 57 |
| n4,g6 | 2 | 29 |
| n6 | 1 | 79 |
| n9 | 1 | 55 |
| n7 | 1 | 81 |
| n3e | 1 | 26 |
| n3a | 1 | 20 |
| NeuNAc | 3 | 100 |
| GalNAc | 3 | 20 |

^a^ The division of the raw integral by the number of protons assumes they all contribute equally, which is unknown and unlikely.

^b^ Some of the peak overlap cannot be deconvoluted. In some cases, e.g. n8/n9, the peaks are strongly coupled and so not independent.

**Supplementary Table 3.** (**a**) Primers used to amplify VH and VL of TKH2. (**b**) Primers used to generate alanine mutants in TKH2. In the primer name, F stands for forward primer, R stands for reverse primer.

| **a.** Primers used to amlify TKH2 VH and VL | | | |
| --- | --- | --- | --- |
| **Primer #** | **Primer name** | **Primer sequence 5🡪3** |  |
| 1 | 5′ MsVHE | GGGAATTCGAGGTGCAGCTGCAGGAGTCTGG |  |
| 2 | 3′ Cγ1 outer | GGAAGGTGTGCACACCGCTGGAC |  |
| 3 | 5′ L-Vκ_3 | TGCTGCTGCTCTGGGTTCCAG |  |
| 4 | 5′ L-Vκ_4 | ATTWTCAGCTTCCTGCTAATC |  |
| 5 | 5′ L-Vκ_5 | TTTTGCTTTTCTGGATTYCAG |  |
| 6 | 5′ L-Vκ_6 | TCGTGTTKCTSTGGTTGTCTG |  |
| 7 | 5′ L-Vκ_6,8,9 | ATGGAATCACAGRCYCWGGT |  |
| 8 | 5′ L-Vκ_14 | TCTTGTTGCTCTGGTTYCCAG |  |
| 9 | 5′ L-Vκ_19 | CAGTTCCTGGGGCTCTTGTTGTTC |  |
| 10 | 5′ L-Vκ_20 | CTCACTAGCTCTTCTCCTC |  |
| 11 | 3′ mCκ | GATGGTGGGAAGATGGATACAGTT |  |
| 12 | 5′ mVλ1/2 | CAGGCTGTTGTGACTCAG |  |
| 13 | 5′ mVλx | CAACTTGTGCTCACTCAG |  |
| 14 | 3′ mCλ outer | GTACCATYTGCCTTCCAGKCCACT |  |
| 15 | 3′ Cγ1 inner | GCTCAGGGAAATAGCCCTTGAC |  |
| 16 | 5′ AgeI P-mVH09 | CTGCAACCGGTGTACATTCCGAGGTGCAGCTGCAGGAGTCTGG |  |
| 17 | 3′ SalI P-mJH03 | TGCGAAGTCGACGCTGCAGAGACAGTGACCAGAG |  |
| 18 | 5′ AgeI P-mVK03 | CTGCAACCGGTGTACATTCCCAAATTGTTCTCACCCAGTCTCCA |  |
| 19 | 3′ BsiWI P-mJK04 | GCCACCGTACGTTTCAGCTCCAGCTTGGTC |  |

| **Primer #** | **Primer name** | **Primer sequence 5🡪3** |
| --- | --- | --- |
| **b.** Primers used to generate alanine mutants in TKH2 | | |
| 1 | Anti-AcSTn-heavy-S31AF | CTCATTAATCGCCTATGGTGTAAGCTGGG |
| 2 | Anti-AcSTn-heavy-S31AR | AACCCTGAGACAGTGCATG |
| 3 | Anti-AcSTn-heavy-Y32AF | ATTAATCAGCGCTGGTGTAAGCTGGG |
| 4 | Anti-AcSTn-heavy-Y32AR | GAGAACCCTGAGACAGTG |
| 5 | Anti-AcSTn-heavy-G33AF | ATCAGCTATGCTGTAAGCTGGG |
| 6 | Anti-AcSTn-heavy-G33AR | TAATGAGAACCCTGAGAC |
| 7 | Anti-AcSTn-heavy-V50AF | TGGCTGGGAGCAATATGGGGTG |
| 8 | Anti-AcSTn-heavy-V50AR | CTCCAGACCCTTTCCTGG |
| 9 | Anti-AcSTn-heavy-W52AF | GGGAGTAATAGCGGGTGACGGGAG |
| 10 | Anti-AcSTn-heavy-W52AR | AGCCACTCCAGACCCTTT |
| 11 | Anti-AcSTn-heavy-G53AF | GTAATATGGGCTGACGGGAGCACAAATTATC |
| 12 | Anti-AcSTn-heavy-G53AR | TCCCAGCCACTCCAGACC |
| 13 | Anti-AcSTn-heavy-R96AF | TGTCGGACCCGCGTTTGCTTACTG |
| 14 | Anti-AcSTn-heavy-R96AR | CAGTAGTACGTGGCTGTG |
| 15 | Anti-AcSTn-light-S31AF | CTCAAGTGTAGCTTACATGCACTGGTACCAGC |
| 16 | Anti-AcSTn-light-S31AR | CTGGCACTGCAGGTCATG |
| 17 | Anti-AcSTn-light-Y32AF | AAGTGTAAGTGCCATGCACTGGTACCAG |
| 18 | Anti-AcSTn-light-Y32AR | GAGCTGGCACTGCAGGTC |
| 19 | Anti-AcSTn-light-H34AF | AAGTTACATGGCCTGGTACCAGCAGAAG |
| 20 | Anti-AcSTn-light-H34AR | ACACTTGAGCTGGCACTG |
| 21 | Anti-AcSTn-light-D50AF | TGGATTTATGCCACATCCAAACTGACTTC |
| 22 | Anti-AcSTn-light-D50AR | TCTTTTGGGGGAGGTGCC |
| 23 | Anti-AcSTn-light-Q89AF | TTATTACTGCGCGCAGTGGAGTAGTAATACG |
| 24 | Anti-AcSTn-light-Q89AR | GTGGCAGCATCTTCAGCC |
| 25 | Anti-AcSTn-light-Q90AF | TTACTGCCAGGCGTGGAGTAGTAATAC |
| 26 | Anti-AcSTn-light-Q90AR | TAAGTGGCAGCATCTTCAG |
| 27 | Anti-AcSTn-light-W91AF | CTGCCAGCAGGCGAGTAGTAATAC |
| 28 | Anti-AcSTn-light-W91AR | TAATAAGTGGCAGCATCTTC |
| 29 | Anti-AcSTn-light-S92AF | CCAGCAGTGGGCTAGTAATACGCTC |
| 30 | Anti-AcSTn-light-S92AR | CAGTAATAAGTGGCAGCATC |
| 31 | Anti-AcSTn-light-S93AF | GCAGTGGAGTGCTAATACGCTCACG |
| 32 | Anti-AcSTn-light-S93AR | TGGCAGTAATAAGTGGCAG |
| 33 | Anti-AcSTn-light-N94AF | GTGGAGTAGTGCTACGCTCACG |
| 34 | Anti-AcSTn-light-N94AR | TGCTGGCAGTAATAAGTG |
| 35 | Anti-AcSTn-light-L96AF | TAGTAATACGGCCACGTTCGGTGC |
| 36 | Anti-AcSTn-light-L96AR | CTCCACTGCTGGCAGTAA |
